# Boron Enrichment in Martian Clay

**DOI:** 10.1371/journal.pone.0064624

**Published:** 2013-06-06

**Authors:** James D. Stephenson, Lydia J. Hallis, Kazuhide Nagashima, Stephen J. Freeland

**Affiliations:** 1 NASA Astrobiology Institute, University of Hawaii, Honolulu, Hawaii, United States of America; 2 Hawaii Institute of Geophysics and Planetology, University of Hawaii, Honolulu, Hawaii, United States of America; NIGMS, NIH, United States of America

## Abstract

We have detected a concentration of boron in martian clay far in excess of that in any previously reported extra-terrestrial object. This enrichment indicates that the chemistry necessary for the formation of ribose, a key component of RNA, could have existed on Mars since the formation of early clay deposits, contemporary to the emergence of life on Earth. Given the greater similarity of Earth and Mars early in their geological history, and the extensive disruption of Earth's earliest mineralogy by plate tectonics, we suggest that the conditions for prebiotic ribose synthesis may be better understood by further Mars exploration.

## Introduction

The sugar ribose is central to metabolism, most notably as the derivatized sugar component of RNA. Any theory of life’s origins focused on RNA must therefore include a plausible prebiotic ribose production pathway [1], [2]. Borate minerals have been shown to stabilize ribose [3], [4] synthesized via the formose reaction [5], making boron a potentially important chemical element connecting geoscience to organic chemistry. One of the main objections to this mode of ribose accumulation on the early Earth is that evaporitic borate deposits (e.g. colemanite, ulexite and kernite) may not have been present on the early Earth (>3.5 Ga) [6]. Our research suggests boron-enriched clay as an alternative site for ribose production.

**Figure 1:**
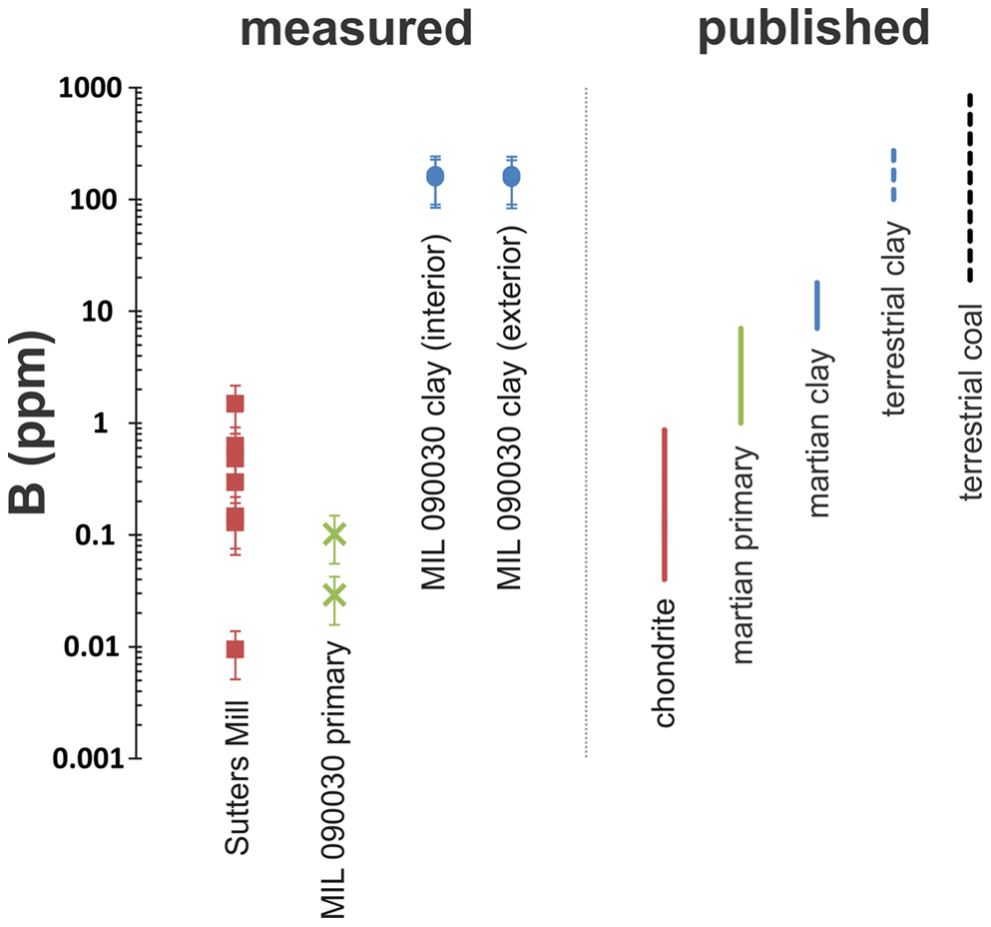
Boron abundance values measured in this study compared with previously published values. Comparison data was from chondrite meteorite phases, martian meteorite primary and alteration phases (including clays), terrestrial clays and coals. Measured data from the Sutter’s Mill chondrite and from martian meteorite MIL 090030 show similar or reduced boron concentrations to their previously measured equivalents. The degree of boron enrichment in MIL 090030 clay data is most closely comparable to terrestrial clays, it is highly enriched compared to other phases in the same meteorite and to previously measured martian clays. Error bars are 2σ.

Clays have long been proposed as excellent locations for prebiotic catalysis [7], [8], polymerization [9], [10], and compartmentalization [11] because of their ability to absorb and protect necessary reactants [12]. These properties are evident in the popularity of clays as catalysts within industry [13]. Boron is commonly concentrated as borate (BO_3_^3−^ or BO_4_^3−^) in terrestrial clays and organic-rich sediments (∼80–800 ppm), but it has never been found at concentrations above 20ppm in any extraterrestrial source (Figure 1, Table S1). Here we use secondary ion mass spectrometry to show that Earth-like boron concentrations exist in martian clay.

## Results and Discussion

Martian meteorite MIL 090030 was collected in the Miller Range region of Antarctica during the 2009/10 ANSMET field season. It belongs to the nakhlite group of martian meteorites - basaltic lavas that crystallized ∼1.3 billion years ago [14], [15]. Subsequent to crystallization these meteorites were aqueously altered on Mars, which produced narrow (generally <100 µm wide) alteration veins containing evaporite salts, amorphous silicate, Fe-oxides and smectite clays [16]–[19]. We determined the abundance of boron in the alteration veins of MIL 090030, via in-situ Cameca ims 1280 ion-microprobe analyses at the University of Hawaii (Materials and Methods). For comparison we also analyzed the primary igneous minerals of MIL 090030, as well as various primary and aqueous alteration phases in Sutters Mill – a carbonaceous chondrite meteorite observed to fall in 2012 [20].

The abundances of boron measured in Sutter’s Mill olivine and matrix material (Table 1) are comparable to data previously reported for boron in the carbonaceous chondrites (0.004–0.7 ppm) [21]. Furthermore, boron abundances in the igneous primary minerals (olivine and pyroxene) of MIL 090030 are comparable to those of the carbonaceous chondrites (Table 1). In contrast, boron is concentrated in martian alteration veins (∼160 ppm), to levels rivaling those found in terrestrial clays and marine sediments (Figure 1). The level of boron concentration in the alteration veins of MIL 090030 cannot be explained by terrestrial contamination, as no atmospheric reservoir on Earth approaches the abundance of boron measured in these alteration veins (Figure 1). However, as smectite clays and amorphous materials can readily adsorb atmospheric and aqueous contaminants, both an exterior (MIL090030,25) and interior (MIL 090030,23) sample from the MIL 090030 meteorite stone were measured. We found no measureable difference between boron abundances in the alteration veins of the internal and external areas, implying terrestrial boron is negligible. In addition, heavy pre-sputtering prior to each ion-probe analysis (Figure S1 and Figure S2) removes any surface contamination which may result from sample preparation (Materials and Methods). Therefore, the boron concentration in MIL 090030 alteration veins must be the result of secondary alteration processes on Mars.

**Table 1:** Measured sample and standard data.

Sample	Boron (ppm)	2σ error
Sutters Mill olivine	0.01	0.004
Sutters Mill matrix 1	1.49	0.68
Sutters Mill matrix 2	0.48	0.22
Sutters Mill matrix 3	0.63	0.29
MIL 090030,23 olivine 1	0.03	0.01
MIL 090030,23 pyroxene 1	0.10	0.05
MIL 090030,23 alteration vein 1	156	72
MIL 090030,23 alteration vein 2	166	76
MIL 090030,23 alteration vein 3	155	71
MIL 090030,25 alteration vein 1	154	71
MIL 090030,25 alteration vein 2	166	76
BCR glass 1	5.1	2.3
BCR glass 2	4.7	2.2
BCR glass 3	5.0	2.3
BCR glass 4	4.9	2.2
BCR glass 5	5.4	2.5
BCR glass 6	4.9	2.2
BCR glass 7	5.3	2.4
GSA glass 1	27	12
GSA glass 2	28	13
GSA glass 3	32	15
GSA glass 4	31	14

Boron is readily adsorbed onto clay surfaces [22], [23], and has a greater affinity for smectite and illite than for other clay species [24]–[26]. Given that MIL 090030 alteration veins contain smectite it follows that boron enrichments should be found in these veins. However, the degree of enrichment reported here is somewhat surprising - an order of magnitude higher than any other extraterrestrial phase investigated to date [20], [27], [28].

A straightforward geochemical interpretation of our results is that boron, a relatively volatile and soluble element, was first concentrated in the fluid dregs of lava (4–7 ppm boron has been detected in the late stage mesostasis of other nakhlites [28]) and then became further concentrated by any groundwater or hydrothermal fluids that came into contact with the rock. Due to the oxidizing conditions of martian clay the boron most likely exists as borate, possibly in isolation or else bound to cations such as calcium, magnesium or sodium.

Borates may be crucial to prebiotic chemistry due to their ability to stabilize ribose, a key component of RNA. Without them, ribose degrades, reducing it to only a small fraction of the formose reaction products [29]–[31]. With borate ribose can last months. This results in a relative enrichment of ribose and other aldopentoses [32] compared to other formose products [3], . Clays have long been implicated as ideal catalytic surfaces for prebiotic reactions [7], hence borate enriched martian clay may represent a potential site for ribose synthesis.

MIL090030 offers a glimpse into the chemistry of Mars at the time of its clay’s formation, but coupled with other data it also offers some insight into the potential of earlier clays. Remote sensing observations of the martian surface [33], [34] suggest an early (>3.7 Ga) wet environment, compared to the later, relatively dry conditions under which the MIL 090030 clays formed. During this earlier and wetter clay-forming period Fe-smectite clays similar to those found in MIL 090030 were dominant [34]. Hence, there is no reason to assume the earlier clays were comparatively boron depleted. Gale crater, which is currently being explored by the Curiosity rover, contains Fe-smectite units within its layered deposits [35], which may have formed in a lake environment on early Mars (3.6–3.8 Ga) [36].

This old (>3.7 Ga), relatively wet period in Mars history is contemporaneous with current estimates for the origin of life on Earth. Similar clay deposits to those produced in this wet martian environment appear to have formed on the early Earth as well. For example, intensively studied ancient (3.8 Ga) tourmaline grains from western Greenland appear to have formed from boron-rich clay precursors [37], [38]. Given the greater similarity of Earth and Mars early in their geological history, further exploration of Mars may prove invaluable in answering how ribsose may have accumulated on the Earth prebiotically.

## Materials and Methods

### Sample Preparation and Petrographic Analysis

We were allocated two thin-sections of the nakhlite martian meteorite MIL 090030 by CAPTEM, prepared in 2011 at the NASA Johnson Space Center. MIL 090030,25 was deliberately cut from the external surface of the ∼450 g parent meteorite, whereas MIL 090030,23 was taken from the central portion. One thin-section (#51) of the Sutter’s Mill meteorite, which was seen to fall in California in 2012, was allocated to UH by Q.-Z. Yin at UC Davis. This section of Sutter’s Mill has been identified as a CM2.0/CM2.1-type carbonaceous chondrite [20].

We utilized the JEOL JSM-5900LV scanning electron microscope at the University of Hawaii (UH) to produce whole-thin-section backscatter-electron images and elemental X-ray images at low resolution (20 µm pixel size), in order to locate areas of interest. These areas were subsequently X-ray imaged at higher resolutions, with various pixel sizes (1–5 µm), to determine the best places for ion microprobe analyses. To verify the positions of the sputtered regions, these areas were also imaged after analyses.

The major- and minor-element chemistry of each phase was determined via JEOL JXA-8500F electron microprobe (EMP) analyses at UH (Table S2). The analytical conditions of the electron microprobe included a 10 nA primary beam current, 15 keV accelerating voltage, and 5 µm spot size. The count time for beam-sensitive elements (e.g., Na and K) was set at 15 s, whereas that for less sensitive elements was set at 30 s. Beam-sensitive elements were measured first. Matrix effects were corrected using PAP procedures. The elemental detection limits are 0.03 wt.% for SiO_2_, Al_2_O_3_, and MgO; 0.04 wt.% for TiO_2_, CaO, and K_2_O; 0.06 wt.% for Na_2_O and Cr_2_O_3_; 0.07 wt.% for MnO; 0.08 wt.% for FeO. San Carlos olivine (NMNH 111312) and Rockport fayalite (USNM 85276) were utilized as standards, along with NMNH 117075 chromite. UH standardized orthoclase Or-1 (5–168), Lake County labradorite, Kakanui augite, Amelia Albite, Sphene glass and Verma garnet were also used as standards.

All three of these thin-sections were prepared using epoxy resin. EMP analyses suggest this resin contains trace amounts of boron (<0.01 wt %). Therefore, all areas close to epoxy were carefully avoided as potential sites for boron abundance analysis. None of our analysis areas contain epoxy within the pre-sputtered region, as evidenced by X-ray imaging.

### Boron Abundance Analyses

The ions of boron (^11^B^+^), calcium (^40^Ca^+^) and magnesium (^26^Mg^+^) in both standard materials and unknowns were measured in situ using the UH Cameca ims 1280 ion microprobe, with a primary ^16^O^−^ ion beam accelerated to 13 keV and an impact energy of 23 keV. Positively charged secondary ions were accelerated to +10 keV and an energy window of 50 eV was used. ^40^Ca and ^26^Mg were measured to provide internal standardization for calcium and magnesium rich phases, respectively (Equation 1 and Equation 2) – the true wt % values of these elements for each analysis area were measured via electron microprobe analysis, as discussed above. To minimize possible surface contamination of boron, a 10nA focused ^16^O^−^ primary ion beam was rastered over 30×30 µm area for 1000 seconds. The data were collected with a 1nA focused primary beam rastered over 10×10 µm area at the center of the pre-sputtered area (Figure S1, Figure S2). The count rates of ^11^B^+^ during measurements did not show systematic decrease with time and were typically constant within their errors, indicating that the pre-sputtering procedure completely removed any surface contamination. The mass resolving power was ∼2000, sufficient to separate any interfering molecules. ^11^B^+^ was measured on an electron multiplier while ^26^Mg^+^ and ^40^Ca^+^ were measured by a Faraday cup. Each measurement consisted of 30 cycles, with ^11^B measured for 20s, ^26^Mg for 2s, and ^40^Ca for 2s during each cycle. The background count rate of the electron multiplier is ∼0.002 cps. Under these conditions, the detection limit of B was less than 1 ppb [39].

Data were corrected for sensitivities among B, Ca, and Mg using USGS basaltic glass standards BCR-2G and GSA-1G, containing 6±1 ppm and 23±7 ppm boron, respectively (from GeoRem data base, [40]). BCR-2G was prepared by melting kilogram aliquots of (powdered) BCR-2 (Columbia River basalt), BHVO-2 (Hawaiian Volcanic Observatory basalt), and BIR-1 (Icelandic basalt), at 1540°C under a nitrogen atmosphere. GSA-1G was prepared using a set of synthetic glass powders - it has an overall basaltic composition but is doped with more than fifty trace elements at concentrations of <0.1 µg g^−1^. Repeat analyses of these standards allow for the calculation of sensitivity factor (Equation 1). Both ^26^Mg and ^40^Ca are abundant in the glass standards, and so either could be used to calculate sensitivity factor, in Equation 1 ^26^Mg is used as an example:
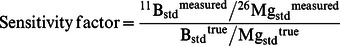
(1)


The sensitivity factor, in turn, allows for the calculation of the concentration of B in the unknown Equation 2. Again, depending on the composition of the unknown, either ^26^Mg or ^40^Ca was used. We used ^26^Mg for most phases as it was most abundant in olivine, pyroxene, the martian alteration veins (containing the smectite clay), and the matrix of Sutter’s Mill. However, we did use ^40^Ca for calcite and dolomite.
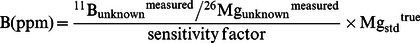
(2)


The sensitivity factors obtained for the two basaltic glass standards have a systematic difference of ∼40–50% (Table S3). The reported errors include this uncertainty.

Our boron analysis methodology is similar to that reported by [41] who used glass standards to determine boron abundance in smectite clays. These authors reported matrix effects to be unnoticeable. As discussed in detail by [42], matrix affects between glasses and crystalline phases are small (<10%) when Si is used for normalization. If Mg or Ca are used instead of silicon (as in this study), these affects are reduced further.

## Supporting Information

Figure S1 **Secondary electron images of Cameca ims 1280 ion microprobe analysis pits in Sutters Mill.** The extent of the 30 µm pre-sputtering raster is clearly visible as a slightly lighter grey area around the central 10 µm analysis pit of olivine (olv) 1 (A). Due to their smaller grain size, the pre-sputtering raster goes beyond the boundaries of calcites 1 and 2, and dolomite 1, but the central pit is within these carbonates (B–D). The heterogeneous nature of the matrix in Sutter’s Mill makes it more difficult to distinguish the extent of the analysis pits (E–H). However, our data suggest that despite this heterogeneity the matrix is relatively uniform with respect to boron.
(PDF)

Figure S2 **Secondary electron images of Cameca ims 1280 ion microprobe analysis pits in MIL 090030,23 (A–F) and MIL 090030,25 (G–H).** As in Figure S1, the pre-sputtered 30 µm region in these images is visible as a slightly lighter grey area surrounding the central 10 µm analysis pit (labels mark the extent of this area). For alteration vein analyses the pre-sputtered area extends beyond the margins of the vein. However, all central analysis pits lie within these margins. MIL 090030,25 alteration vein 1 exhibits weathered olivine around its margins. All visible cracks are empty of epoxy resin (as evidenced by X-ray imaging). Olv = olivine, cpx = clinopyroxene.
(PDF)

Table S1 **Previously published boron reservoir data.**
(PDF)

Table S2 **Representative major and minor element abundances.**
(PDF)

Table S3 **Glass standard measured boron abundances.**
(PDF)

Text S1 **Supporting references for Table S1.**
(PDF)
