# Supplementary material for: Boron Enrichment in Martian Clay
Source: PLoS One. 2013 Jun 6;8(6):e64624. doi: 10.1371/journal.pone.0064624 (PMC3675118; doi:10.1371/journal.pone.0064624)
Supplement: Figure S1 — Secondary electron images of Cameca ims 1280 ion microprobe analysis pits in Sutters Mill. The extent of the 30 µm pre-sputtering raster is clearly visible as a slightly lighter grey area around the central 10 µm analysis pit of olivine (olv) 1 (A). Due to their smaller grain size, the pre-sputtering raster goes beyond the boundaries of calcites 1 and 2, and dolomite 1, but the central pit is within these carbonates (B–D). The heterogeneous nature of the matrix in Sutter’s Mill makes it more difficult to distinguish the extent of the analysis pits (E–H). However, our data suggest that despite this heterogeneity the matrix is relatively uniform with respect to boron. (PDF) [file pone.0064624.s001.pdf]

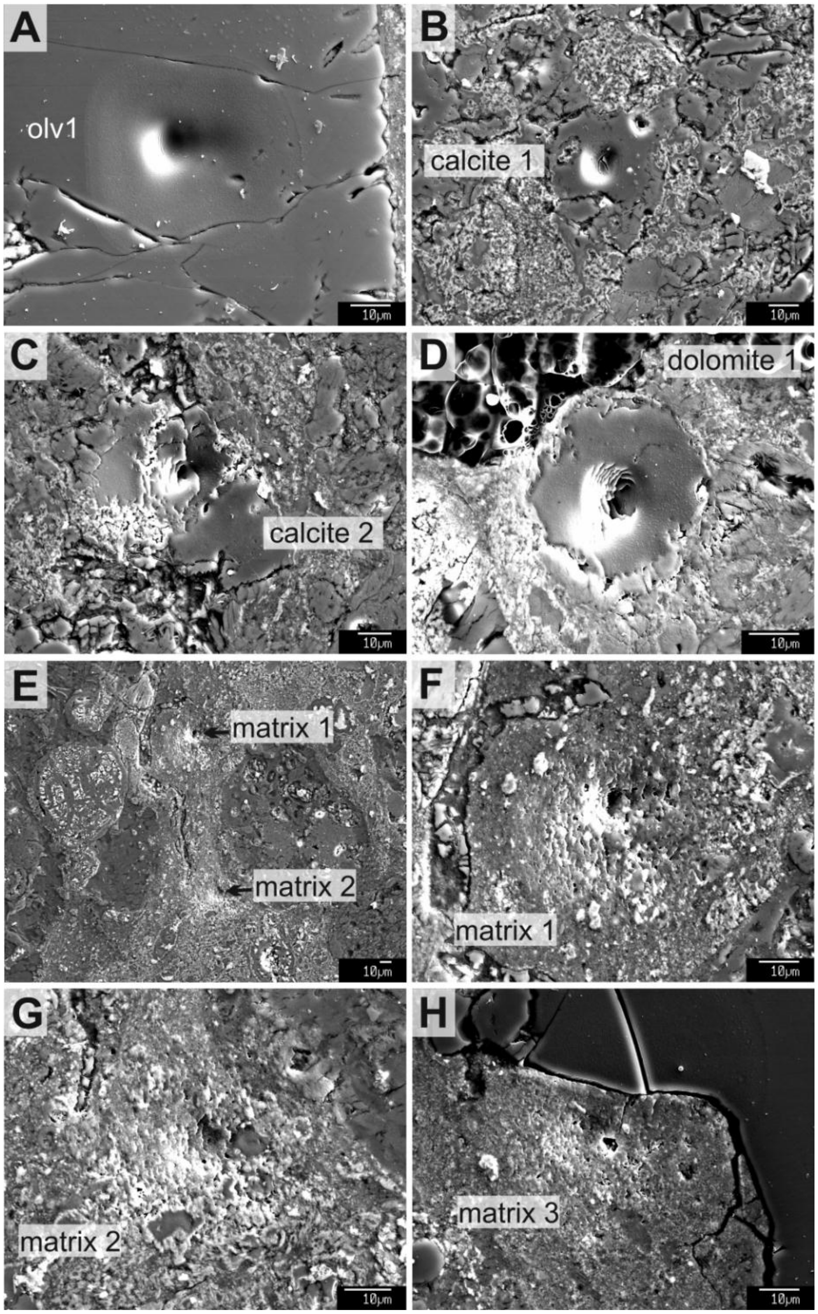

Figure S1: Secondary electron images of Cameca ims 1280 ion microprobe analysis pits in Sutters Mill. The extent of the 30 µm pre-sputtering raster is clearly visible as a slightly lighter grey area around the central 10 µm analysis pit of olivine (olv) 1 (**A**). Due to their smaller grain size, the pre-sputtering raster goes beyond the boundaries of calcites 1 and 2, and dolomite 1, but the central pit is within these carbonates (**B-D**). The heterogeneous nature of the matrix in Sutter's Mill makes it more difficult to distinguish the extent of the analysis pits (**E-H**). However, our data suggest that despite this heterogeneity the matrix is relatively uniform with respect to boron.
