# Supplementary material for: Boron Enrichment in Martian Clay
Source: PLoS One. 2013 Jun 6;8(6):e64624. doi: 10.1371/journal.pone.0064624 (PMC3675118; doi:10.1371/journal.pone.0064624)
Supplement: Figure S2 — Secondary electron images of Cameca ims 1280 ion microprobe analysis pits in MIL 090030,23 (A–F) and MIL 090030,25 (G–H). As in Figure S1, the pre-sputtered 30 µm region in these images is visible as a slightly lighter grey area surrounding the central 10 µm analysis pit (labels mark the extent of this area). For alteration vein analyses the pre-sputtered area extends beyond the margins of the vein. However, all central analysis pits lie within these margins. MIL 090030,25 alteration vein 1 exhibits weathered olivine around its margins. All visible cracks are empty of epoxy resin (as evidenced by X-ray imaging). Olv = olivine, cpx = clinopyroxene. (PDF) [file pone.0064624.s002.pdf]

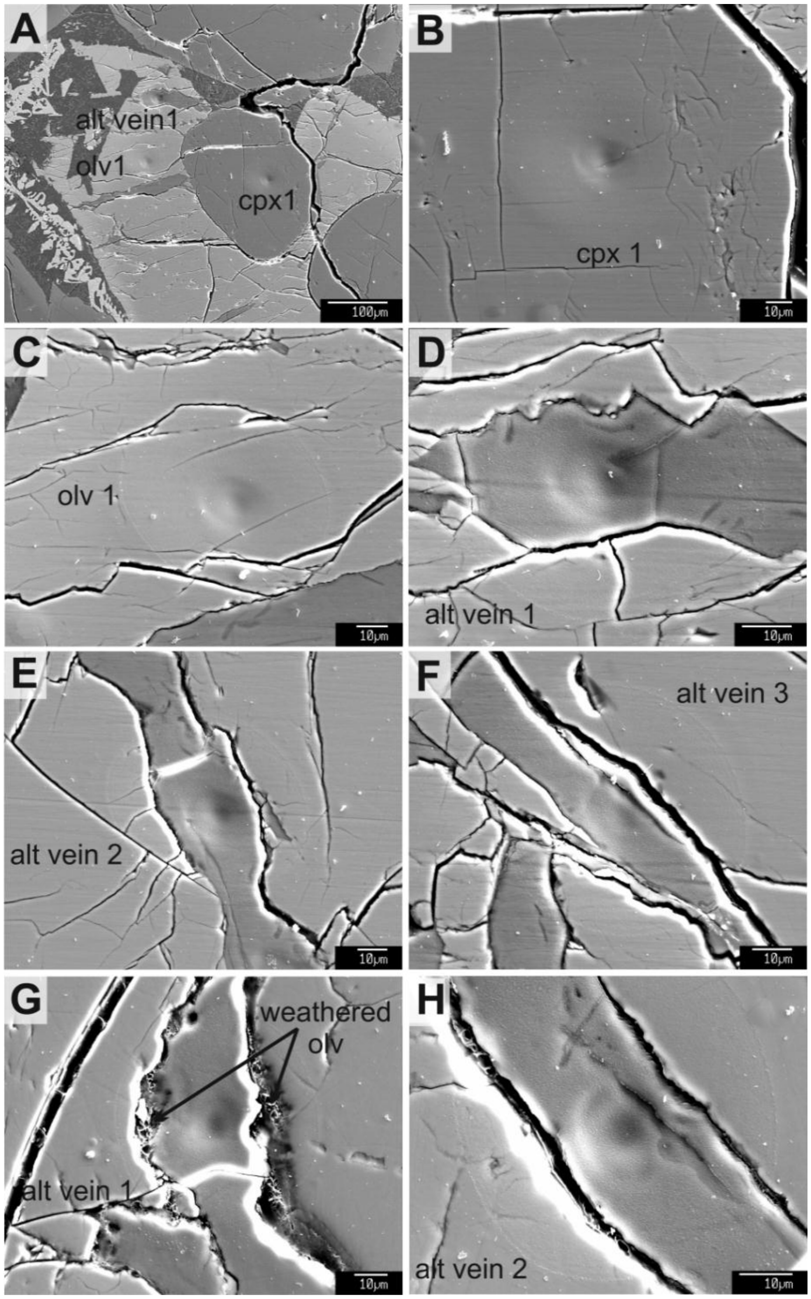

Figure S2: Secondary electron images of Cameca ims 1280 ion microprobe analysis pits in MIL 090030,23 (**A-F**) and MIL 090030,25 (**G-H**). As in Figure S1, the pre-sputtered 30 μm region in these images is visible as a slightly lighter grey area surrounding the central 10 μm analysis pit (labels mark the extent of this area). For alteration vein analyses the pre-sputtered area extends beyond the margins of the vein. However, all central analysis pits lie within these margins. MIL 090030,25 alteration vein 1 exhibits weathered olivine around its margins. All visible cracks are empty of epoxy resin (as evidenced by X-ray imaging). Olv = olivine, cpx = clinopyroxene.
