# Supplementary material for: Boron Enrichment in Martian Clay
Source: PLoS One. 2013 Jun 6;8(6):e64624. doi: 10.1371/journal.pone.0064624 (PMC3675118; doi:10.1371/journal.pone.0064624)
Supplement: Table S1 — Previously published boron reservoir data. (PDF) [file pone.0064624.s003.pdf]

Table S1: Previously published boron reservoir data.

Previously published boron reservoir data	Boron (ppm)
**Earth**	
Bulk silicate Earth (excluding core)	0.3 [43]
Precipitation (water/ice)	0.0001-0.003
Ocean water	4.5 [44]
Basaltic rocks	0.34-1.3 [45]
Clays (smectite and illite) and marine sediments	100-300 [46]
Organic-rich matter (coal)	19-843 [47]
**Chondrite meteorites**	
Bulk CI chondrites	0.87 [27]
Melilite in CAI's (from Efremovka and Allende)	0.04-0.7 [20]
**Mars**	
Bulk silicate Mars	0.6*
Pyroxene (from Nakhla and Lafayette)	< 1 [48]
Mesostasis (from Nakhla)	4 to 7 [28]
Clay ("iddingsite" from Nakhla and Lafayette)	7 to 18 [28,49]

*The condensation temperature of boron is only 100K less than that of potassium. Therefore, based on its volatility, boron, like potassium and phosphorous, should be around twice as abundant on Mars as on Earth.
