# Supplementary material for: Boron Enrichment in Martian Clay
Source: PLoS One. 2013 Jun 6;8(6):e64624. doi: 10.1371/journal.pone.0064624 (PMC3675118; doi:10.1371/journal.pone.0064624)
Supplement: Table S2 — Representative major and minor element abundances. (PDF) [file pone.0064624.s004.pdf]

Table S2: Representative major and minor element abundances.

	SM olivine	SM calcite	SM dolomite	SM matrix 1	SM matrix 2	SM matrix 3	MIL cpx 1	MIL olv 1	MIL alt 1	MIL alt 2	MIL alt 3
SiO_2	42.48	0.03	0.26	26.95	24.16	25.57	51.22	33.03	43.18	41.92	39.35
TiO_2	0.08	bd	0.05	0.04	0.10	0.02	0.37	0.01	0.08	0.08	bd
Al_2O_3	0.28	0.03	0.01	2.29	2.46	2.31	0.88	bd	0.15	0.10	0.13
Cr_2O_3	0.06	0.03	0.04	1.13	0.73	0.26	0.33	bd	bd	bd	0.03
FeO	0.49	1.63	3.47	22.65	30.18	31.08	13.74	46.74	32.64	33.77	32.17
MnO	bd	0.19	1.81	0.19	0.07	0.23	0.40	0.98	0.62	0.60	0.56
MgO	56.60	1.26	17.19	18.89	15.25	13.37	12.71	17.69	5.88	5.92	5.47
CaO	0.69	53.25	30.50	0.20	0.12	0.98	19.33	0.45	0.13	0.18	0.14
Na_2O	0.01	0.01	0.04	0.60	0.61	0.58	0.22	bd	0.01	0.03	0.04
K_2O	bd	bd	bd	0.08	0.06	0.04	bd	bd	0.04	0.04	0.05
CO_2	n/a	43.86	46.50	n/a	n/a	n/a	n/a	n/a	n/a	n/a	n/a
**Total**	100.70	100.28	99.86	73.01	73.72	74.44	99.20	98.90	82.74	82.62	77.96

cpx = clinopyroxene. olv = olivine. alt = alteration vein. bd = below detection. n/a = CO_2 was stoichiometrically calculated for calcite and dolomite, but is not present in the other phases. Low totals for matrix and alteration veins reflect the presence of water and some oxidized iron (Fe_2O_3) – as these are both non-stoichiometric they cannot be accurately calculated.
