# Supplementary material for: Boron Enrichment in Martian Clay
Source: PLoS One. 2013 Jun 6;8(6):e64624. doi: 10.1371/journal.pone.0064624 (PMC3675118; doi:10.1371/journal.pone.0064624)
Supplement: Table S3 — Glass standard measured boron abundances. (PDF) [file pone.0064624.s005.pdf]

Table S3: Glass standard measured boron abundances.

Standard	Boron (ppm)	2σ error
BCR glass 1	5.1	2.3
BCR glass 2	4.7	2.2
BCR glass 3	5.0	2.3
BCR glass 4	4.9	2.2
BCR glass 5	5.4	2.5
BCR glass 6	4.9	2.2
BCR glass 7	5.3	2.4
GSA glass 1	27	12
GSA glass 2	28	13
GSA glass 3	32	15
GSA glass 4	31	14
