# Supplementary material for: Boron Enrichment in Martian Clay
Source: PLoS One. 2013 Jun 6;8(6):e64624. doi: 10.1371/journal.pone.0064624 (PMC3675118; doi:10.1371/journal.pone.0064624)
Supplement: Text S1 — Supporting references for Table S1. (PDF) [file pone.0064624.s006.pdf]

Text S1: Supporting references for table S1.

1. McDonough WF, Sun S-s (1995) The composition of the Earth Chem Geol 120: 223-253.
2. Spivak AJ, Edmond JM (1987) Boron isotope exchange between seawater and the oceanic-crust. Geochim Cosmochim Acta 51: 1033–1043.
3. Chaussidon M, Jambon A (1994) Boron content and isotopic composition of oceanic basalts: Geochemical and cosmochemical implications. Earth Planet Sci Lett 121: 277-291.
4. Goldschmidt VM, Peters C (1932) Geochemie des Bors. I Nachr Ges Wiss Math-physik K1: 402–407.
5. Williams LB. Hervig, RL (2004) Boron isotope composition of coals: a potential tracer of organic contaminated fluids. Appl Geochem 19: 1625–1636.
6. Spivak-Birndorf LJ, Wadhwa M, Williams, LB (2008) Paper presented at the Ground Truth From Mars workshop.
7. Spivak-Birndorf LJ, Wadhwa M, Williams LB (2008) Paper presented at the 39th Lunar Planet Sci Conf.
